# A Standard Milk

**Published:** 1909-11-27

**Authors:** 


### A STANDARD MILK.

Man is becoming more and more the creature of artificial conditions. The crude environment of Nature in which he delights, and amidst which he struggled when the world was younger, recedes with each step of his advance as a civilised being. For his rudimentary animal requirements he has endeavoured to preserve a natural satisfaction, but artifice has invaded and is gradually supplanting the natural indulgence of even his most primitive needs. With each departure from the wont and custom of his pristine state, he has encountered, in the substituted *régime*, agencies inimical to his well-being; but effort guided by improved intelligence has enabled him to adapt to his requirements the sophisticated products which have replaced the natural provision. Whether ultimately this change will prove physically and spiritually beneficial may be an open question, but that it has been rigorously imposed upon him by inevitable force of circumstance cannot be questioned. The standardisation of his requirements and of the modicum of their satisfaction is the measure of his social progress, for in his civil state a nice economy may be said to be the goal of his ambition. In the control and organisation of the food supplies of our urban populations this trend has manifestly been toward securing a minimum standard of quality in articles of diet which should satisfy physiological and hygienic requirements, and in no particular item of food has the difficulty of meeting this irreducible demand been greater than in the case of milk. Natural milk in the fullest sense of the phrase is so variable, so unsatisfactory, so dangerous and even deadly, that it has for long been the despair of the sanitarian. Yet in a more restricted and precise meaning it is a food of such primary importance as to be universally recognised as indispensable.

To secure a normal milk, fresh from the healthy cow, clean, free from adventitious contaminations of greater or less danger to health, produced, handled, transmitted, and distributed to the consumers under sanitary conditions, has for long been the aim of all who have had a real concern in seeing established an irreproachable milk supply. But it has to be confessed that signal failure has attended this most praiseworthy yet withal enormous and most painstaking effort. It is true that here and there a milk has been marketed of unexceptional quality and history, but the problem of a satisfactory public milk supply has remained unsolved.

On Saturday, the 20th inst., there was opened by Lady Lytton, at Knebworth in Herts, a dairy which would seem at last to offer a prospect of solving the vexed problem. Since it would seem impracticable at once to transform our agricultural population, their farms, animals, utensils, habits, and all that makes for the imperfection of their products, the British White Cross Milk Company have set themselves, with such imperfect conditions as must unfortunately for long continue, to provide a milk which shall satisfy all but what the most exacting counsel of perfection can call for. If it be not possible to prevent the admission of dirt to milk—and at present it is not—the next best thing is to remove it as promptly and effectually as possible; and this the White Cross Dairy Company do within an hour of milking. If the freedom of milk from the admission of pathogenic organisms cannot absolutely be ensured—and this, without reforms which under the most sanguine anticipations it must take years to accomplish, it cannot be—then their removal or destruction must be effected with the least delay; and this also is satisfactorily obtained by the company. In a word, a clean milk of constant quality free from injurious organisms or their toxins, and from preservatives, uninjured in its taste and in its chemical or physiological properties, is aimed at, and so far as we can judge is produced, at the dairy at Knebworth.

The British White Cross Milk Company provides for the guarantees it gives by an elaborate system of purification. The means by which this is accomplished is thus officially described. It is maintained that upon all farms, with few exceptions, some manure and other dirt are sure to get into the milk. Under the White Cross system the milk as received from the cow is passed through a centrifugal separator by which all solid particles of dirt are removed, and here the skim milk is separated from the cream. The skim milk is then placed in a tank, *i.e.* a concentrator which is heated to 140° F. by a current of hot water circulating in an outer jacket. Rapid evaporation is next caused by a vigorous current of air drawn through a thick layer of cotton, which passing through the milk produces a violent agitation. When the milk is reduced in this way to a fifth of its bulk it is taken from the concentrator, the pasteurised cream is returned to it, and the resulting mixture is then rapidly cooled down to 40° F. The concentration of the skim milk is in this way determined to a point representing one-fourth of the bulk of the original milk.

It is claimed for this process that all milk so treated is absolutely pure, being free from every particle of dirt and filth which is always present to some extent in other milk prepared for sale under existing conditions. Ordinary milk obtained from a dairy is by no means a uniform product.

It is claimed for White Cross milk that the amount of fat present is always the same, *i.e.* 3.25 per cent. of butter fat, and this amount will never vary. The saving in transportation charges owing to the reduction in bulk, it is stated, enables the White Cross Company to supply this milk to consumers at the current market price of the day.

The process pursued by this company, by removing all bacteria at the source of the milk, prevents the increase of bacteria and the development of toxins. Briefly the White Cross claim amounts to this, that White Cross milk, though derived originally from the same source as ordinary milk, is purer and better than any milk on the market, because it "has been cleaned and deprived of its various odours, and its disease germs have been killed." Possessing this exceptional purity, White Cross milk has neither been sterilised by a high temperature nor preserved by the addition of other more harmful preservatives. It is so identical with the original milk, for it is without a boiled taste or any foreign flavour, and is also free from the impaired digestibility that sterilised milk produces, being, in fact, simply milk as pure as it is humanly possible to make it. It differs from all other milk in that nothing has been added to it, and that it is no more expensive than the least pure milk available for public consumption.

A milk possessing all the advantages which we believe are rightly claimed for White Cross milk, obtainable at the ordinary price of milk, is an unquestionable boon to the milk-consuming public. It is not, in so far as in some measure it is a manufactured product, the milk which it has been the aim of the public health service to secure, but in that it guarantees those qualities which a hygienic milk should possess, it supplies a need the urgency of which will condone the manipulatory means which have prestalled the more natural product of a practice of perfection.

